# The effects of polyphenols against oxidative stress in *Caenorhabditis elegans* are determined by coexisting bacteria

**DOI:** 10.3389/fnut.2022.989427

**Published:** 2022-12-01

**Authors:** Begoña Ayuda-Durán, Eva Sánchez-Hernández, Susana González-Manzano, Celestino Santos-Buelga, Ana M. González-Paramás

**Affiliations:** ^1^Grupo de Investigación en Polifenoles (GIP-USAL), Universidad de Salamanca, Salamanca, Spain; ^2^Department of Agricultural and Forestry Engineering, ETSIIAA, University of Valladolid, Palencia, Spain

**Keywords:** gut microbiota, epicatechin, quercetin, probiotics, *Lactobacillus*, *Bifidobacterium*, *Enterococcus*

## Abstract

**Introduction:**

Increasing evidence supports the role of gut microbiota in many aspects of human health, including immune, metabolic and neurobehavioral traits. Several studies have focused on how different components of the diet, such as polyphenols, can modulate the composition and function of the gut microbiota leading to health benefits.

**Methods:**

The effects on the resistance against thermally induced oxidative stress of *C. elegans* grown in the presence of flavonoids (quercetin or epicatechin) and fed different probiotic strains, namely *Lactobacillus plantarum* CLC17, *Bifidobacterium longum* NCIMB 8809 and *Enterococcus faecium* CECT 410, were explored.

**Results:**

Feeding *C. elegans* with the assayed bacteria in the absence of flavonoids did not significantly affect body size and fertility of the worms neither improve their resistance against oxidative stress compared to *E. coli* controls. However, increased resistance to stress was found when *C. elegans* was cultivated in the presence of both *L. plantarum* and flavonoids, but not with *B. longum* or *E. faecium*. An exploratory study revealed the presence of glycosylated and sulfated metabolites together with the aglycone in worms treated with quercetin and fed any of the different assayed LAB strains. However, in the assays with epicatechin a differential metabolite, tentatively identified as 5-(4′-hydroxyphenyl)-γ-valerolactone 3′-*O*-glucoside, was detected in the worms fed *L. plantarum* but not with the other bacteria.

**Conclusion:**

The obtained results indicated that the interactions bacteria/polyphenol play a key role in the effects produced in *C. elegans* regarding resistance against oxidative stress, although those effects cannot be only explained by the ability of bacteria to metabolize polyphenols, but other mechanisms should also be involved.

## Introduction

Probiotic bacteria have been linked with beneficial effects on the health of the host (1). Several clinical studies have attributed to probiotics a high therapeutic potential in the treatment of diarrhea associated with antibiotics (2), inflammatory bowel disease (3), obesity and diabetes (4), respiratory tract infections in children (5), anti-carcinogenic effects (6) and prevention of urinary tract infections (7). Probiotics may exert their action on the host in different ways, including modulation of the composition of the intestinal microbiota, maintenance of the integrity of the intestinal barrier or improvement of the human immune system (8, 9). The most commonly used probiotic microorganisms are Lactic Acid Bacteria (LAB) such as *Lactobacillus*, *Bifidobacterium* and *Enterococcus* species, the latter harboring both pathogenic and commensal microorganisms, although only in rare occasions they are associated with a risk of infection for humans (10).

*Lactobacillus plantarum*, one of the most important species of the genus *Lactobacillus*, has been reported to decrease cholesterol levels and reduce symptoms of gastrointestinal disorders (11). Different strains of *L. plantarum* have been shown to be able to metabolize polyphenols, exerting antimicrobial activity against a wide range of pathogenic bacteria, owing to their ability to produce low molecular weight compounds with bactericidal potential (12, 13). In particular, *L. plantarum* CLC17 strain, isolated from breast milk (14), can degrade polyphenols to a variety of end products of phenolic catabolism, like syringic, vanillic, 3-*O*-methyl gallic and gallic acids, being its growth in turn stimulated by the presence of phenolic compounds and their metabolites (15). Furthermore, this strain can provide protection to the intestinal ecosystem, reducing the content of bacteria belonging to the *Enterobacteriaceae* family (16). These effects have led to postulate *L. plantarum* CLC17 to be used as a food ingredient to improve the metabolism of dietary polyphenols (16). The genus *Bifidobacterium* possesses recognized ability to metabolize dietary carbohydrates and is believed to allow efficient and persistent colonization in the gut (17). *Bifidobacterium longum* subsp. *longum*, commonly found in the human gut microbiota and with perceived positive health effects (18), also demonstrated capable of metabolizing hydroxycinnamic acids (19). The administration of *B. longum* has been proposed to contribute to prevent gastrointestinal disturbances such as diarrhea, constipation and bowel cancer (6). *Enterococcus* strains are highly adapted to various food systems due to their good tolerance to salts and acids, being involved in cheese and sausage fermentation processes (20). Different strains of *E. faecium* and *E. faecalis* have been proposed as probiotics (21), being shown to exhibit desirable features, such as high adhesion and low cytotoxicity, as well as cytoprotective and anti-inflammatory properties in *in vitro* studies (22). In particular, *E. faecium* CECT 410 strain was reported to be able to colonize pig intestinal microbiota and to reduce coliforms in their feces (23).

The phenolic compounds, commonly referred to as polyphenols, are a group of plant secondary metabolites widespread in higher plants, where they contribute to the mechanisms of natural resistance. They are also widely distributed in the human diet through grains, fruits and vegetables, and derived products, such as wine, tea or chocolate, in which they contribute to sensory, technological and health properties. Based on their chemical structures, they can be divided into different subgroups. Among them, flavonoids constitute the largest group in nature, being broadly distributed in the human diet. The consumption of a diet rich in flavonoids has been associated with a decreased incidence of the most common chronic diseases in developed countries, like type 2 diabetes, different types of cancer, neurodegenerative illnesses and cardiovascular disorders (24).

Most dietary polyphenols are not absorbed by the small intestine and reach the gut, where they interact with the intestinal microbiota to be metabolized to a range of metabolites, some of which can be bioactive and contribute to the potential health effects associated to polyphenol consumption. The composition of the intestinal microbiota varies among people, which may affect the metabolism of the ingested polyphenols (25). For their part, polyphenols and their metabolites might influence the composition and function of the gut microbiota acting on their growth or metabolism, as well as avoiding the growth of pathogens (25, 26). Actually, phenolic compounds have been attributed prebiotic properties associated with their antimicrobial activity and capacity to modulate the intestinal microbiota (27). Thus, two-way interactions between polyphenols and microbiota could contribute to their putative effects in human health.

In order to clarify the interactions polyphenols-microbiota, the use of simple organisms like *C. elegans* can be very useful. This worm is a free-living bacterivore that grows optimally in a wide variety of microorganisms. Zhang et al. (28) denoted significant differences in the worm’s microbial populations depending on the area and environment where they have grown. However, in the laboratory the nematode is fed routinely with *Escherichia coli* strain OP50 in a monoxenic culture. This and the synchronization of cultures by bleaching make worms to lose their innate microbiota. Several studies have explored feeding the nematode with different bacterial strains. Sánchez-Blanco et al. (29) found that *C. elegans* fed *Bacillus subtilis* (PY79, 3610, and 168 strains), present in its usual soil habitat, lived longer (43–58%) than those fed the standard *E. coli* diet. The authors showed that this difference was not due to the nutritive quality of the diet, but to factors contributed by the bacterium, namely the presence of coenzyme Q (CoQ) synthesized by *E. coli* but not by *B. subtilis*. The authors proposed that CoQ supplementation provided by *E. coli* would alter the worm cellular redox homeostasis leading to a decreased longevity, exemplifying the relevance of the microbiome on life expectancy. Regarding LAB, it has been observed that *C. elegans* fed heat-killed *L. fermentum* LA12 and *L. plantarum* CJLP133 presented longer lifespan compared with *E. coli* controls (30). Similarly, worms cultured with *L. salivarus* FDB89 improved the lifespan by a caloric restriction mechanism (31), while *E. faecium* L11 increased worm longevity triggering the expression of genes related to aging and innate immunity in *C. elegans* (32). Feeding with *L. rhamnosus* improved worm resistance against inflammation and oxidative stress (33), whereas *B. animalis* subsp. *lactis* CECT 8145 increased oxidative stress resistance in *C. elegans* and decreased worm body fat and triglycerides (34).

Some studies have focused on the ability of LAB to protect *C. elegans* from pathogenic bacteria, such as *Salmonella enterica* serovar Enteritis (35), *Salmonella enterica* serovar Typhimurium (32), *Yersinia enterocolitica* (36), *Legionella pneumophila* (37), *E. coli* (38), and *Staphylococcus aureus* and *E. coli* O157:H7 (39). Quite recently, Kumar et al. (40), employed *C. elegans* to explore the probiotic potential of a *Lactobacillus plantarum* strain (LPJBC5). Those authors concluded that this strain was able to increase worm lifespan, improve stress resistance and promote a series of traits associated with healthy aging, such as physical and cognition performance, fat accumulation, gut integrity, or mitochondrial function. The observed effects were suggested to be mediated by the activation of the p38 MAPK signaling pathway and downstream targets, such as the SKN-1 transcription factor, upregulating the expression of stress resistance genes. Furthermore, LPJBC5 was also able to downregulate *fat-5* and *fat-7* genes modulating fat metabolism, as well as to upregulate genes involved in serotonin signaling (*ser-1, tph-1*, and *mod-1*) related to improved cognitive function (40).

The aim of the present work is to evaluate the ability of three LAB strains, *Lactobacillus plantarum* CLC17, *Bifidobacterium longum* NCIMB 8809 and *Enterococcus faecium* CECT 410, to modulate *C. elegans* resistance against oxidative stress by themselves and when combined with polyphenols. Epicatechin and quercetin, belonging to the flavan-3-ol and flavonol classes, respectively, and that are two of the best-represented flavonoids in the human diet, were employed as model polyphenols.

## Materials and methods

### Standards and reagents

(-)-Epicatechin (EC), quercetin (Quer), ampicillin sodium salt, nistatine, agar, yeast extract, cholesterol, 5-fluoro-2′-deoxyuridine (FUdR), and bovine serum albumin were purchased from Sigma-Aldrich (Madrid, Spain). Sodium chloride, calcium chloride, 10% w/v sodium hypochlorite solution, hydrogen chloride, sodium hydroxide and dimethyl sulfoxide (DMSO) were obtained from Panreac (Barcelona, Spain). Potassium dihydrogen phosphate, potassium monohydrogen phosphate, sodium monohydrogen phosphate, acetic acid and magnesium sulfate were from Merck (Darmstadt, Germany). Petri plates Ø 35 and 60 mm were from Brand GMBH (Wertheim, Germany) and Petri plates Ø 90 mm from Francisco Soria Melguizo (Valdemoro, Madrid). MRS Broth was from Fisher Scientific (Madrid, Spain) and tryptone medium from Fluka Analytical (Madrid, Spain).

### Strains and maintenance conditions

The *C. elegans* wild-type strain N2 and *E. coli* OP50 bacterial strain were obtained from the Caenorhabditis Genetics Center at the University of Minnesota (Minneapolis, USA). Probiotic strains *L. plantarum* CLC17, *B. longum* NCIMB 8809 and *E. faecium* CECT 410 were provided by the Department of Food Biotechnology and Microbiology of the Food Science Research Institute (CIAL-CSIC, Madrid, Spain).

LAB strains were cultured in De Man, Rogosa, and Sharpe (MRS) broth supplemented with L-cysteine (0.05%) at 37°C for 24 h in anaerobic conditions. In order to be used as standard feed for nematodes, *E. coli* OP50 was grown in LB broth at 37°C for 24 h. Bacteria were collected by centrifugation at 16,300 *g* for 1 min, washed twice with sterile M9 buffer (3 g KH_2_PO_4_, 6 g Na_2_HPO_4_, 5 g NaCl, 1 ml 1 M MgSO_4_, H_2_O to 1 L) and centrifuged again at 16,300 *g* for 1 min to remove the supernatant. The bacteria were adjusted to a final concentration of 0.10 mg (wet weight) per μL in M9 buffer. The suspended cells were placed on nematode growth medium (NGM) plates and dried.

EC and Quer solutions (200 mM) in DMSO were added to the nematode growth medium during its preparation to get a 200 μM final concentration of the flavonoids on the plates. Control plates were also prepared without the compounds but containing the same volume of DMSO (0.1% DMSO, v/v).

Wild worms were routinely propagated at 20°C on NGM plates containing *E. coli* OP50 as a food source. Synchronization of worm cultures was achieved by treating gravid hermaphrodites with sodium hypochlorite-sodium hydroxide solution 5 N (50:50). Eggs are resistant whereas worms are dissolved in the bleach solution. The suspension was vortex shaken for one min, kept a further minute on rest and then centrifuged (2 min, 8,700 *g*); this process was repeated five times. The final pellet containing the eggs was washed six times with an equal volume of buffer M9. A volume of 100–300 μL of the M9 buffer with eggs (depending on eggs concentration) was transferred and incubated on NGM agar plates in the presence of the flavonoids (EC or Quer) or in their absence. The synchronized L1 larvae were fed with *E. coli* OP50 until they reached the larval state L4. Then, they were moved to new plates covered with a lawn of each of the different bacteria, i.e., *E. coli* OP50, as a control, *L. plantarum* CLC17, *B. longum* NCIMB 8809, or *E. faecium* CECT 410, and supplemented or not with EC or Quer. The worms were transferred every 2 days to fresh plates until they reached the day of the assay. In all cases, the plates also contained FUdR at a concentration of 150 μM to prevent reproduction and progeny overgrowth.

### Body length measurement

Age-synchronous worms were grown to the L4 stage on NGM plates seeded with *E. coli* OP50, then transferred individually to plates containing either OP50 or the probiotic strain and maintained at 20°C for 6 days. Nematode length was measured every 24 h alive with a microscope (Leica M205 FA, Germany) equipped with a camera (Leica DFC 420), coupled with a Leica Application Suite V3 data processing software. The images were analyzed using the Image J software. Nematode length was measured in triplicate from about 10 individuals per treatment and assay.

### Reproduction assays

Synchronized L4 worms fed *E. coli* OP50 were individually transferred to plates with the different bacterial strains and stored at 20°C for 6 days until their reproductive stage ceased. During that time, the worms were moved every day to fresh plates with the corresponding assayed bacteria. The offspring of each animal was counted to verify that eggs were fertile. For each of the studied LAB, assays were performed three times with 10 worms per assay.

### Thermal stress assays

L1 nematodes were incubated on OP50 plates with or without the assayed flavonoid. At the L4 stage, the nematodes were placed onto the different bacterial lawns (*L. plantarum* CLC17, *B. longum* NCIMB 8809, *E. faecium* CECT 410 or *E. coli* OP50), and, in the assays with the dual bacteria/flavonoid system, also in the presence of EC or Quer. Worms from the different treatments were transferred with a platinum wire to agar plates (Ø 35 mm, 20 worms per plate) at two stages of development: reproduction (2nd day of adulthood) and post-reproduction stage (9th day of adulthood), at which they were submitted to thermally induced oxidative stress (35°C, 8 h). Afterward, the number of dead and alive nematodes was counted. The relative rates of survival of worms after being subjected to thermal stress were expressed in relation to the untreated controls. Assays were carried out with approximately 100 nematodes per treatment, performing three independent trials per assay.

### Assessment of flavonoids uptake by *Caenorhabditis elegans*

Synchronized worms were grown until L4 stage with OP50 in the presence or not of the assayed flavonoids. Then, animals were washed twice with M9 to remove the *E. coli* as much as possible and moved to fresh plates that contained *E. coli* or the different bacteria (*L. plantarum* CLC17, *B. longum* NCIMB 8809, *E. faecium* CECT 410) with or without the flavonoid (EC or Quer). After 4 days, the worm biomass was washed successively once with M9, three times with PBST (PBS + 0.01% Tween 20), once with PBS and finally with M9 buffer.

The remaining worm pellet was resuspended in 1 ml of 30% methanol, the mixture was vortex shaken vigorously and further homogenized in a Thermo Savant FastPrep 120 Cell Disrupter System at a speed of 5.5 m/s using 7 cycles of 10 s. The supernatant was then collected and the pellet washed and centrifuged further two times with 1 ml of 30% methanol. The combined methanolic supernatants were dried in a centrifugal concentrator micVac (GeneVac, Ipswich, UK) and the residue dissolved in 500 μL of acetonitrile 20% for high-performance liquid chromatography (HPLC)-DAD/MS analysis. Two independent experiments were performed.

Total protein was also determined by the Bradford method after digestion of the worm homogenates, in order to express compound concentration in relation to worm protein.

### Analysis by high-performance liquid chromatography-diode array detection-mass spectrometry

Analyses were performed in a Hewlett-Packard 1100 chromatograph (Agilent Technologies, Waldbronn, Germany) equipped with a quaternary pump and a diode array detector (DAD) coupled to an HP Chem Station (rev. A.05.04) data-processing station. A Waters Spherisorb S3 ODS-2C8, 3 μm (4.6 × 150 mm) column thermostatted at 30°C was used. The solvents used were: (A) 0.1% formic acid, and (B) acetonitrile. The elution gradient was 100% A to 15% B in 35 min, and from 15 to 40% B over 10 min, at a flow rate of 0.5 ml min^–1^. Double online detection was carried out in the DAD at 280 nm and in a mass spectrometer (MS) connected to the HPLC system via the DAD cell outlet. MS detection was performed in an API 3200 Qtrap (Applied Biosystems, Darmstadt, Germany) equipped with an ESI source and a triple quadrupole-ion trap mass analyzer, which was controlled by the Analyst 5.1 software. Zero grade air served as the nebulizer gas (30 psi) and as turbo gas (400°C) for solvent drying (40 psi). Nitrogen was used as the curtain (20 psi) and collision gas (medium). Both quadrupoles were set at unit resolution. The ion spray voltage was operated at –4,500 V in the negative mode. Method settings were: declustering potential (DP), –40 V; entrance potential (EP), –10 V; collision energy (CE), –50 V; and cell exit potential (CXP) –3 V. Fragmentation of the parent ions was obtained in enhanced product ion (EPI) mode using the following settings: declustering potential (DP), –50 V; entrance potential (EP), –6 V; collision energy (CE), –25 V; and collision energy spread (CES) 0 V.

### Statistical analyses

Statistical analyses were performed using the SPSS PC software package (version 25.0, SPSS Inc., Chicago). ANOVA was used for multiple comparisons of values to determine possible significant differences between the treated and control groups in the phenotypic characterization tests: body length and reproduction. For thermal stress survival trials, contingency tables were made, and statistical significance was calculated using the Chi-Square Test. In each analysis, the differences were considered statistically significant at the level of *p* < 0.05.

## Results and discussion

### Assessment of *Caenorhabditis elegans* viability

Phenotypical characteristics (body length and reproduction rate) of the worms cultured with the LAB bacteria (*L. plantarum* CLC17, *B. longum* NCIMB 8809, and *E. faecium* CECT410) were checked and compared with those fed *E. coli* OP50, in order to test if *C. elegans* viability could be affected by the bacterial strain. It must be noted that cultivation had to be made using *E. coli* OP50 as a feed during the larval stage, and only after reaching the larval state L4 worms were transferred to plates seeded with the different bacteria. This practical approach is required since probiotic bacteria like *Lactobacillus* cause hatched eggs to be arrested as L1 larvae (41).

**FIGURE 1:**
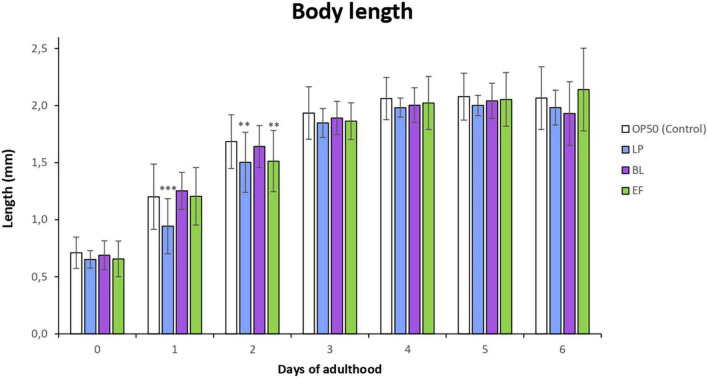
Changes in body length in *C. elegans* fed *Lactobacillus plantarum* CLC17 (LP), *Bifidobacterium longum* NCIMB 8809 (BL), *Enterococcus faecium* CECT 410 (EF) and *E. coli* OP50 (control) from 1st to 6th day of worm adulthood. The error bars represent standard deviation (*n* = 30). Differences in body length in worms fed LAB bacteria vs. *E. coli* OP50 were considered significant at ***p < 0.001, **p < 0.01.

Figure 1 shows the changes in the body length of worms grown in the presence of the different bacteria for 6 days. It was observed that *C. elegans* fed *L. plantarum* and *E. faecium* showed a developmental delay leading to a significantly shorter body length compared to the control (*E. coli* OP50) in the first and second day of adulthood. However, from day 3 onward no significant differences were found in body length among individuals cultivated with the different bacteria. This developmental delay could be explained by the change of food from *E. coli* (larval phase) to LAB strains (L4 onward), as worms need some period of adaptation to the distinct bacteria.

**FIGURE 2:**
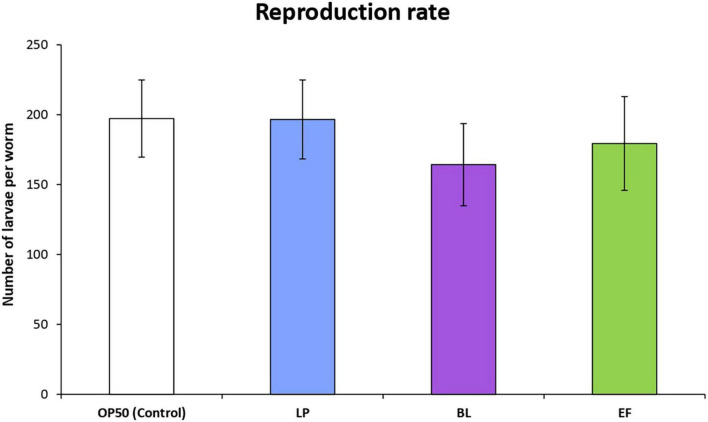
Influence of *Lactobacillus plantarum* CLC17 (LP), *Bifidobacterium longum* NCIMB 8809 (BL), *Enterococcus faecium* CECT 410 (EF) and *E. coli* OP50 (control) in the reproduction rate of *C. elegans.* The error bars represent standard deviation (*n* = 30 worms). No significant differences among worms cultivated with the different bacteria were observed (*p* < 0.05).

The rate of reproduction of *C. elegans* cultivated in the presence of the different assayed bacteria was examined by scoring total progeny eggs from 30 worms for the first 6 days of adulthood. The results are depicted in Figure 2. Cultivation with *B. longum* and *E. faecium* led to a smaller number of viable eggs compared with *E. coli* control, although the differences were no significant. In all cases, worms started to lay eggs the first day of adult and until approximately the fourth day. Nevertheless, in the case of those grown in presence of *B. longum* a delay of some hours at the beginning of reproduction was observed with respect to the *E. coli* OP50 control, which might explain the lower rate of egg production compared with the other bacteria.

Several studies have observed a smaller body size and a reduction in the reproduction capacity in worms fed with different probiotic strains. Schifano et al. (42) found that *Lactobacillus fermentum* MBC2 affected *C. elegans* fertility reducing the reproduction rate of the progeny by 40% compared to *E. coli* OP50 fed animals, and the body length of worms was also shorter. Zhao et al. (31) observed that worms grown in the presence of *L. salivarus* were remarkably smaller and showed a decrease in their reproduction rate compared to those fed *E. coli*, which was explained by a possible reduction in the caloric intake of the worm. Ikeda et al. (35) reported that worms fed *B. infantis* presented a significant decrease in their body size in the first 3 days after the change of food from *E. coli* OP50 to the probiotic to equal the control in the following days, an observation similar to the one made in the present study.

The obtained results confirmed that the assayed LAB can be used to feed *C. elegans* without affecting significantly worm viability and phenotypic and reproductive characteristics. Therefore, we could proceed to check whether LAB could provide advantages on worm resistance against oxidative stress, either by themselves or in combination with polyphenols.

### Assessment of the antioxidant effect of bacterial strains in *Caenorhabditis elegans*

The three assayed probiotic bacteria were assessed to check whether they could influence the resistance of *C. elegans* against oxidative damage. With this aim, worms grown from L4 larval stage in the presence *of L. plantarum* CLC17, *B. longum* NCIMB 8809 or *E. faecium* CECT410 were subjected to thermally induced oxidative stress (35°C, 8 h) applied on the 2nd and 9th day of adulthood and compared to those fed *E. coli* OP50 as a control. These times were chosen to verify if the effects could be different according to the age of the nematode, i.e., young reproductive adults or older adults in post-reproductive stage.

**FIGURE 3:**
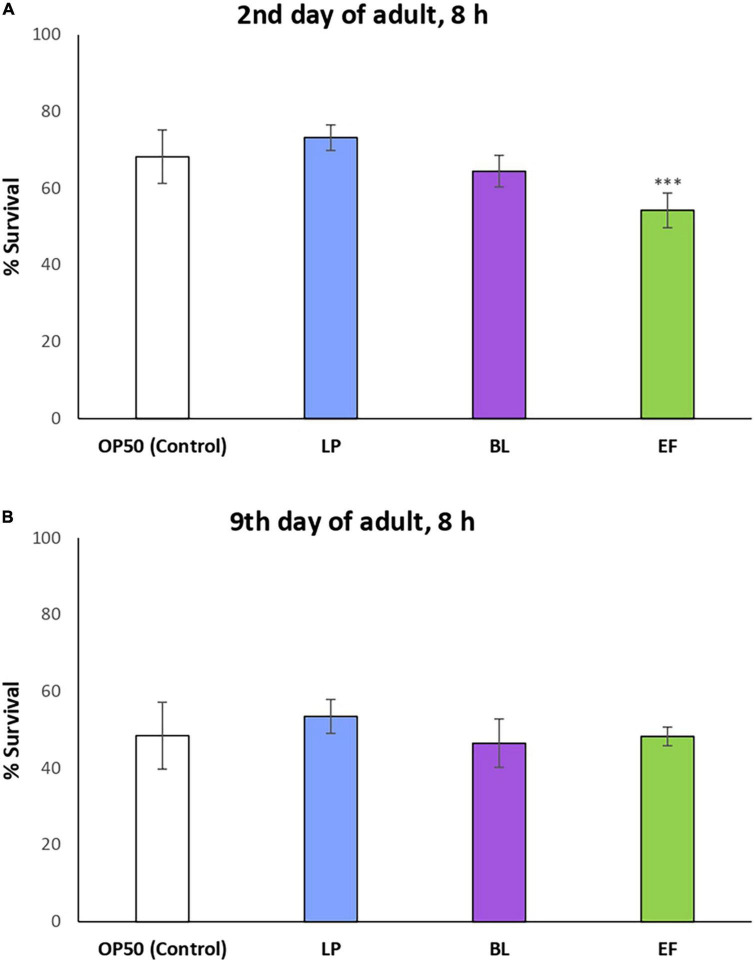
Percentages of survival following thermal stress (35°C, 8 h) applied at days 2 **(A)** and 9 of adulthood **(B)** in N2 wild type *C. elegans* grown in presence of different bacteria strains: *Lactobacillus plantarum* CLC17 (LP), *Bifidobacterium longum* NCIMB 8809 (BL), *Enterococcus faecium* CECT 410 (EF) and *E. coli* OP50 (control). Statistical significance was calculated using the Chi-Square Test. The differences were considered significant at ***p < 0.001.

As it can be seen in Figure 3, despite some variations were observed in the percentages of survival of *C. elegans* fed *L. plantarum* and *B. longum*, the differences among them and with the *E. coli* control were not significant, either at days 2 or 9 of adulthood. By contrast, a significant decrease was produced in the survival of worms cultivated with *E. faecium* CECT410 after the stress applied on day 2, although no differences were found when the stress was applied on day 9. These findings seem coherent with the variation in the body length (Figure 1), which was also decreased at day 2 compared to control worms. None of the assayed bacteria seemed to provide additional advantages against oxidative stress compared to usual *E. coli* feed, and all the effects observed after day 2 could be explained by the adaptation of the worms to a new food source. These results do not agree with those obtained by some authors with other probiotic strains, like *Lactobacillus rhamnosus* CNCM I-3690 (33), *Lactobacillus gasseri* SBT2055 (43), *Bifidobacterium animalis* subsp. lactis CECT 8145 (34) and *Bifidobacterium longum* CECT 7347 (44), all of them providing *C. elegans* enhanced resistance against acute oxidative stress damage.

### Effect of bacteria/polyphenol systems on *Caenorhabditis elegans* viability and resistance to oxidative stress

The effects on resistance to oxidative stress of dual bacteria/polyphenol systems was assessed in worms cultured in the presence of each of the assayed probiotics and two highly consumed flavonoids. In particular, epicatechin (EC), a monomeric flavan-3-ol, and quercetin (Quer), a flavonol, compounds that are widely distributed in the human diet through a variety of fruits, vegetables and derived products, whose consumption has been related to health-promoting effects (24). The assays were carried out using concentrations of EC and Quer in the culture medium of 200 μM, a level that was chosen based on previous studies of our group where a range of concentrations was checked (10–500 μM). In those studies, a hormetic response of *C. elegans* to polyphenols was observed, leading to beneficial effects on longevity and resistance to oxidative stress up to 200 μM, while detrimental effects were produced at higher concentrations (45, 46). Similar results regarding improvement in lifespan and stress resistance were also observed by other authors at that concentration (47, 48).

**FIGURE 4:**
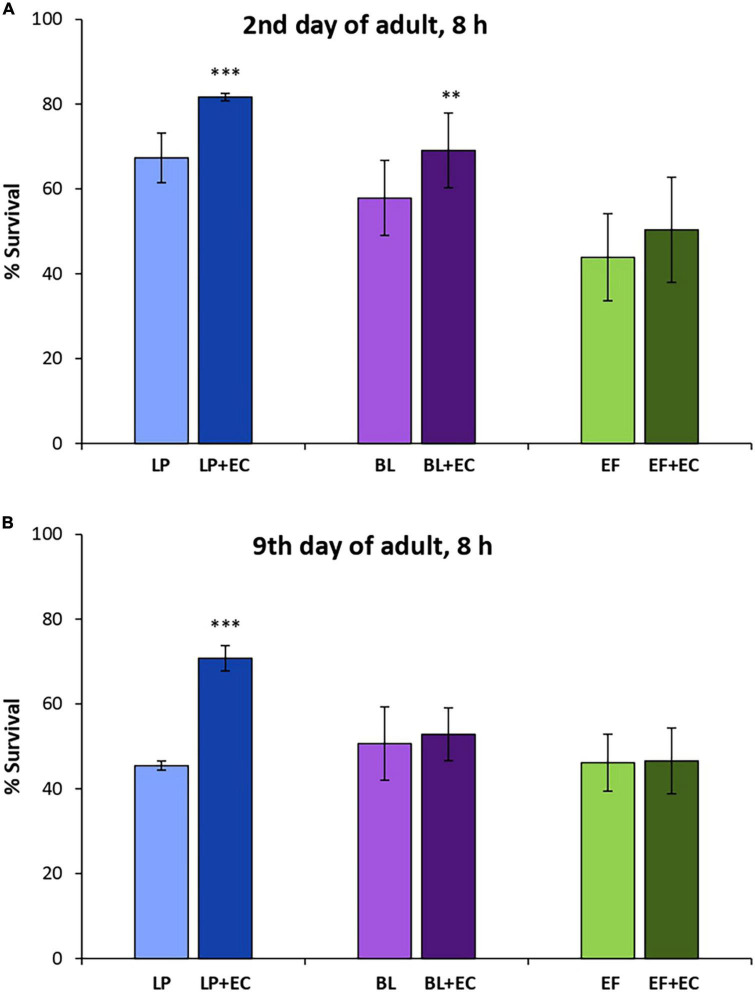
Survival rates of N2 wild type *C. elegans* after being submitted to thermal stress (35°C, 8 h) applied at days 2 **(A)** or 9 of adulthood **(B)** following cultivation with and without epicatechin (EC, 200 μM) in the presence of *Lactobacillus plantarum* CLC17 (LP), *Bifidobacterium longum* NCIMB 8809 (BL) or *Enterococcus faecium* CECT 410 (EF). Differences between worms grown without and with EC were calculated for each strain using the Chi-Square Test. The differences were considered significant at ***p < 0.001 and **p < 0.01.

Significant increases in survival were found in *C. elegans* cultivated with *L. plantarum* and *B. longum* in the presence of EC (200 μM) after being subjected to thermal stress (35°C, 8 h) at day 2 of adulthood compared to those grown with the probiotics alone (Figure 4A). However, in older worms (day 9) the increase was only significant for the system *L. plantarum* + EC but not for *B. longum* (Figure 4B). No significant differences were found in worms fed *E. faecium* at any of the assayed days.

**FIGURE 5:**
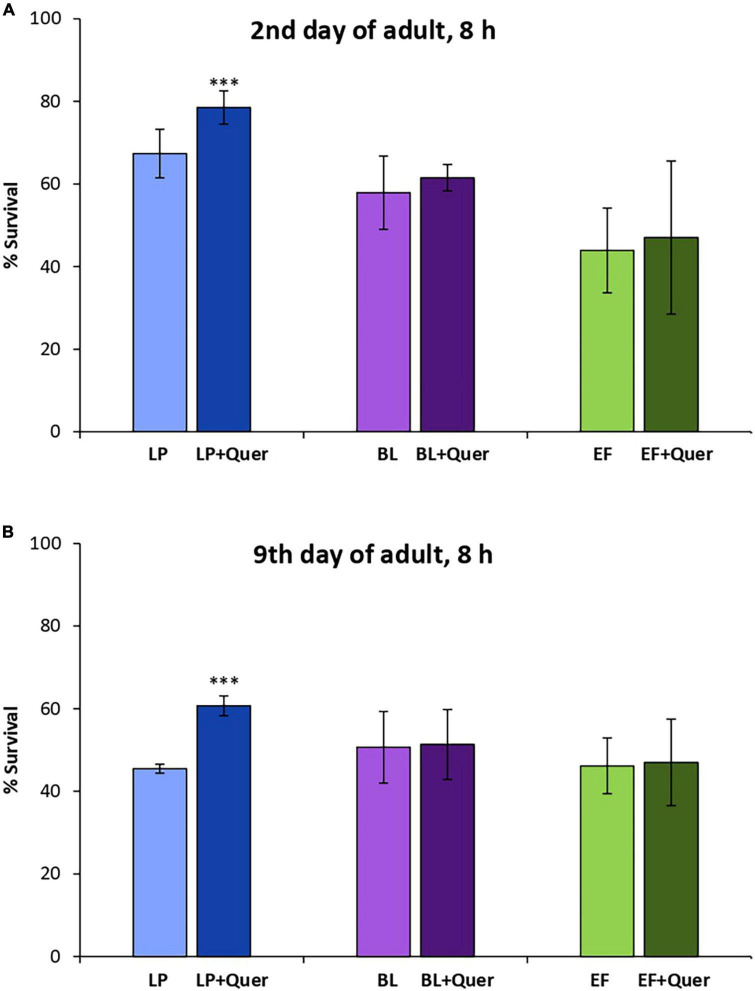
Survival rates of N2 wild type *C. elegans* after being submitted to thermal stress (35°C, 8 h) applied at days 2 **(A)** and 9 of adulthood **(B)** following cultivation with and without quercetin (Quer, 200 μM) in the presence of *Lactobacillus plantarum* CLC17 (LP), *Bifidobacterium longum* NCIMB 8809 (BL) or *Enterococcus faecium* CECT 410 (EF). Differences between worms grown without and with Quer were calculated for each strain using the Chi-Square Test. The differences were considered significant at ***p < 0.001.

Similar observations as for EC were made in the assays carried out in the presence of Quer (200 μM), where significant increases in the survival rate after stress were only observed in worms fed *L. plantarum*, both in young and older animals. In this case, no improvement in the survival was found in the systems *B. longum* + Quer and *E. faecium* + Quer, either in young or older worms (Figure 5).

The results obtained for *L. plantarum* are similar to those previously obtained by our group in studies with *C. elegans* fed *E. coli* OP50, where significant increases in the resistance against thermal and oxidative stress were found in worms cultivated in the presence of EC or Quer (49–51). Positive effects against oxidative stress for these compounds have also been reported by other authors using the canonical *E. coli* OP50 to feed *C. elegans* (47, 52, 53). Similar results were also obtained by our group with other related flavonoids, such as isorhamnetin, tamarixetin, catechin, 4′-O-methylepicatechin and 3′-O-methylepicatechin (49–51, 54).

Different observations were made in the assays with *B. longum*, where positive effects were only observed when combined with EC in young reproductive animals (Figure 4A), but not for Quer neither in older individuals. In the case of *E. faecium* no benefits in the protection against oxidative stress were obtained when combined with EC or Quer (Figures 4, 5).

The results herein obtained with the LAB strains are compared with those previously achieved using *E. coli* OP50 (49–51) in Supplementary Figures 1, 2 included as Supplementary Information. The same experimental conditions have been used in all cases, but in the assays with *E. coli* + EC the stress was applied in days 1 and 10 instead of days 2 and 9, corresponding in either case to young reproductive worms (days 1–2) and non-reproductive adults (days 9–10). In Supplementary Figures 1, 2 it can be verified that the survival rates of *C. elegans* at each development stage are fairly consistent in the current assays with the distinct probiotics and in the previous ones with *E. coli* OP50, despite they were obtained time ago.

### Polyphenols uptake and metabolism

In order to try to evaluate the contribution of the different LAB to flavonoid metabolism, worms were cultured for 4 days in presence of the different bacteria in NGM plates containing Quer, EC or DMSO (control). Afterward, the worm biomass from 2 plates of every assay was collected and homogenized to be analyzed by HPLC-DAD-MS. After a careful study of the complex chromatograms obtained, only those peaks that appeared in any of the treated groups and did not appear in the control group without flavonoids were considered as differential.

The metabolite profile in all worms fed the different bacteria in the assays carried out with Quer was similar in all cases, identifying the quercetin aglycone and some quercetin hexosides and quercetin hexoside sulfates. Although these types of metabolites were unequivocally identified by mass and absorption spectra, it was not possible to quantify them since the concentrations present in the samples were very low and their peaks small, close to the chromatogram baseline and/or overlapped by other peaks. Only the concentration of Quer aglycone could be quantified from the area of their chromatographic peak recorded at 360 nm, and was expressed as μg mg^–1^ of worm protein. The results obtained in the worms fed the different bacteria showed concentrations of Quer aglycone of 0.67 ± 0.10 (OP50), 0.29 ± 0.04 (LP), 4.00 ± 0.44 (BL) and 1.27 ± 0.93 (EF) μg mg^–1^ protein. No direct correlation could be established between these concentrations and the beneficial effects observed regarding resistance to thermal stress in the presence of the different bacteria. It must be indicated that a targeted search was also performed in all MS chromatograms looking for specific masses corresponding to commonly reported quercetin catabolites from degradation by the colonic microbiota, including benzoic, phenylacetic and phenylpropionic acid derivatives. However, none of the searched compounds was specifically detected in the treated worms. In the end, the only clear conclusion is that quercetin was taken up by the worms and that different amounts of this flavonoid were accumulated by them depending on the type of bacteria used for cultivation, although the same concentration of quercetin was present in the plates.

Different observations were made in the studies with EC. Firstly, contrary to Quer, EC and, conjugated EC metabolites (i.e., glycosylated/sulfated derivatives) could not be found in the chromatograms obtained from the assays with any of the different bacteria. This might suggest that a complete degradation of EC was produced, either by worms or bacteria. Despite this, it was apparent is that, in the cases where beneficial effects were produced, they should be explained by the combined system flavonoid + bacteria, as they were not observed in the assays where only bacteria were present. Another relevant observation was that in the case of *L. plantarum* a metabolite from EC degradation could be specifically detected in the MS chromatograms, which was tentatively identified as 5-(4′-hydroxyphenyl)-γ-valerolactone 3′-*O*-glucoside, owing to its previous description as a characteristic metabolite from flavan-3-ol microbiota degradation (55). It is possible that the positive effects observed in worms cultivated in the presence of *L. plantarum* + EC could be related, at least in part, to the apparent ability of this strain to catabolize the flavan-3-ol yielding valerolactones as potential bioactive metabolites, as they were not observed in the assays with *B. longum* and *E. faecalis* strains.

As an example, mass chromatograms obtained for the assays performed with *C. elegans* grown in the presence of *L. plantarum* and Quer or EC, indicating the peaks identified as flavonoid metabolites, are included in Supplementary Figure 3.

The whole of the observations made strongly suggest that the beneficial effects that flavonoids may have in counteracting oxidative damage in *C. elegans* are determined by the type of bacteria. Indeed, if the observed effects were due to the antioxidant activity of the flavonoids, similar results regarding stress resistance should have been expected in the presence of the distinct bacteria. Most of the authors that have studied the possible beneficial effect of LAB in combination with polyphenols attribute the observed benefits to the ability of the bacteria to metabolize the phenolic compounds (12, 13, 15). Our data, especially for Quer, do not provide sufficient basis for concluding that a differential metabolization profile by the distinct bacteria used in our study could exist that might explain the different effects. Nevertheless, although no correlation could be concluded between metabolites and beneficial effects, the obtained results confirm that flavonoids are taken up by *C. elegans* and that the combination of bacteria + polyphenol is determining for the observed effect.

In earlier studies, it was shown that rather than a direct antioxidant effect, the ability of EC and Quer to improve the resistance against oxidative stress in *C. elegans* might involve the modulation of transcription factors and genes in molecular pathways related to the endogenous mechanisms of defense, such as the insulin/IGF-1 signaling and MAPK pathways (48, 50, 51). In a recent paper, Kumar et al. (40) reported that *L. plantarum* LPJBC5 strain was able to increase the lifespan and improve stress resistance in *C. elegans* through the activation of the p38 MAPK signaling pathway, upregulating the expression of stress resistance genes. The results obtained in the present work seem to suggest that not only the potential ability of *L. plantarum* to interfere on key molecular pathways but also its interaction with flavonoids could be determining to interpret the effects observed herein regarding resistance against oxidative stress. This might explain the differential effects observed for this probiotic strain and the other two assayed LAB.

## Conclusion

Feeding *C. elegans* with the assayed LAB was found not to affect significantly the viability of the worm, despite some delay in its growth was observed in the first days of cultivation after changing from *E. coli* to the other bacteria as a food source. Similarly, cultivation in the presence of the probiotics did not modify the resistance of the worm against thermally induced oxidative stress in relation to *E. coli* control. However, an increase in the stress resistance was found when *C. elegans* was cultivated in the presence of both *Lactobacillus plantarum* CLC17 and flavonoids (quercetin or epicatechin), but not in the combination of *Bifidobacterium longum* or *Enterococcus faecium* with the same flavonoids. These results reveal that different bacteria may distinctively affect the way the worm responds to flavonoids, highlighting the importance of considering the interaction bacteria + flavonoid to explain the effects on the resistance to stress, a point that was not evident in the previous assays when only *E. coli* OP50 was used to feed the worm. Indeed, in that case the effect could be interpreted as directly due to the flavonoids. It seems now evident that it is the combined system bacteria + flavonoid that accounts for the effects. On the other hand, the resistance to thermal stress in worms cultivated in presence of *L. plantarum* + EC might be explained by the differential production of potential bioactive metabolites, such as valerolactones, while no clear relationship with any particular metabolite could be established in the assays with Quer. Anyway, what seems evident is that flavonoids are taken up by *C. elegans* and the beneficial effects that they may have in counteracting oxidative damage in the worm are determined by the concomitant presence of particular types of bacteria.

## Data availability statement

The raw data supporting the conclusions of this article will be made available by the authors, without undue reservation.

## Author contributions

CS-B and AG-P: conceptualization, resources, project administration, and funding acquisition. BA-D, SG-M, and ES-H: methodology and formal analysis. BA-D, ES-H, and CS-B: writing—original draft preparation. BA-D, CS-B, and AG-P: writing—review and editing. CS-B, SG-M, and AG-P: supervision. All authors contributed to the article and approved the submitted version.
