# Supplementary material for: The effects of polyphenols against oxidative stress in Caenorhabditis elegans are determined by coexisting bacteria
Source: Front Nutr. 2022 Dec 1;9:989427. doi: 10.3389/fnut.2022.989427 (PMC9752899; doi:10.3389/fnut.2022.989427)
Supplement: Supplementary file 1 [file Data_Sheet_1.docx]

Supplementary Material

# Supplementary Figures and Tables

Figures, tables, and images will be published under a Creative Commons CC-BY licence

## Supplementary Figures


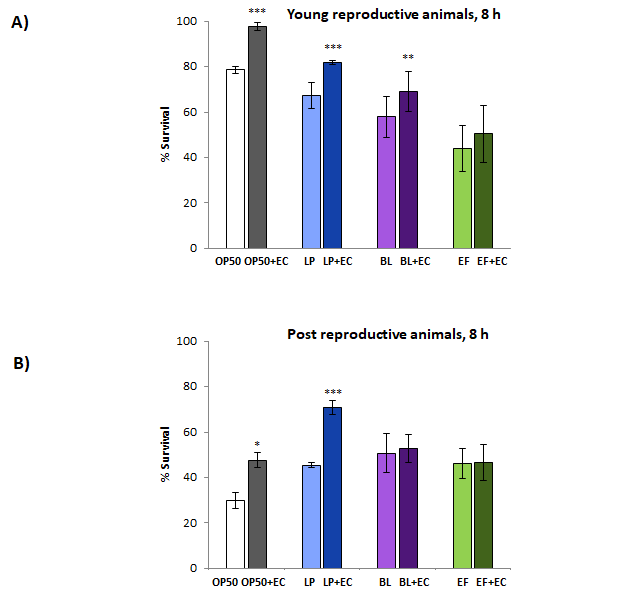


**Supplementary Figure 1.** Survival rates of N2 wild type *C. elegans* after being submitted to thermal stress (35 °C, 8 h) over young reproductive (A) and post-reproductive animals (B) following cultivation with and without epicatechin (EC, 200 μM). Comparison between worms fed *E. coli* OP50 (†) and the different studied bacteria, *Lactobacillus plantarum* CLC17 (LP), *Bifidobacterium longum* NCIMB 8809 (BL) or *Enterococcus faecium* CECT 410 (EF). The stress was applied to worms at days 2 and 9 of adulthood, but in the case of the assays with *E. coli* OP50 performed at days 1 and 10. Differences between worms grown without (controls) and with EC were calculated for each strain using the Chi-Square Test. The differences were considered significant at *** p <0.001, ** p <0.01 and * p <0.05.

(†) The results for the experiments with *E. coli* OP50 were extracted from previous studies (49, 50).

**Supplementary Figure 2.** Survival rates of N2 wild type *C. elegans* after being submitted to thermal stress (35 °C, 8 h) over young reproductive (A) and post-reproductive animals (B) following cultivation with and without quercetin (Quer, 200 μM). Comparison between worms fed *E. coli* OP50 (†) and the different studied bacteria, *Lactobacillus plantarum* CLC17 (LP), *Bifidobacterium longum* NCIMB 8809 (BL) or *Enterococcus faecium* CECT 410 (EF). The stress was applied to worms at days 2 and 9 of adulthood. Differences between worms grown without and with Q were calculated for each strain using the Chi-Square Test. The differences were considered significant at *** p <0.001.

(†) The results for the experiments with *E. coli* OP50 were extracted from previous studies (51)
