# Supplementary material for: The effects of polyphenols against oxidative stress in Caenorhabditis elegans are determined by coexisting bacteria
Source: Front Nutr. 2022 Dec 1;9:989427. doi: 10.3389/fnut.2022.989427 (PMC9752899; doi:10.3389/fnut.2022.989427)
Supplement: Supplementary file 2 [file Data_Sheet_2.docx]

Supplementary Material 2

# Supplementary Figures and Tables

Figures, tables, and images will be published under a Creative Commons CC-BY licence

## Supplementary Figures


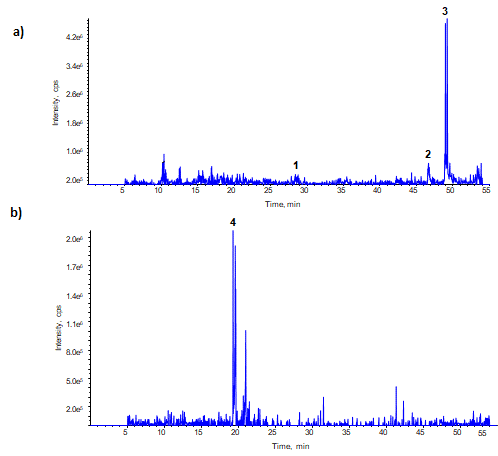


**Figure S3**. a) Extracted ion chromatogram (XIC) for the ion [M−H]^−^ at m/z 301 obtained from homogenized worms fed *Lactobacillus plantarum* treated with quercetin. (b) XIC for the ion [M−H]^−^ at m/z 353 obtained from homogenized worms fed *L. plantarum* treated with EC. The numbers correspond to the compounds tentatively identified as (1) quercetin hexoside sulfate (parent ion [M−H]^−^ at m/z 561), (2) quercetin hexoside (parent ion [M−H]^−^ at m/z 463), (3) quercetin aglycone (parent ion [M−H]^−^ at m/z 301), and (4) 5-(4’-hydroxyphenyl)-γ-valerolactone 3’-O-glucoside (parent ion [M−H]^−^ at m/z 363).
